# Assessment of Microvascular Disturbances in Children with Type 1 Diabetes—A Pilot Study

**DOI:** 10.3390/bios15070439

**Published:** 2025-07-08

**Authors:** Anna Wołoszyn-Durkiewicz, Edyta Dąbrowska, Marcin Hellmann, Anna Jankowska, Mariusz J. Kujawa, Dominik Świętoń, Agata Durawa, Joanna Kuhn, Joanna Szypułowska-Grzyś, Agnieszka Brandt-Varma, Jacek Burzyński, Jędrzej Chrzanowski, Arkadiusz Michalak, Aleksandra Michnowska, Dalia Trzonek, Jacek Wolf, Krzysztof Narkiewicz, Edyta Szurowska, Małgorzata Myśliwiec

**Affiliations:** 1 Department of Pediatrics, Diabetology and Endocrinology, Medical University of Gdansk, 80-211 Gdansk, Poland; joannakuhn91@gmail.com (J.K.); joannaszypulowska@gumed.edu.pl (J.S.-G.); malgorzata.mysliwiec@gumed.edu.pl (M.M.); 2 Center of Translational Medicine, Medical University of Gdansk, 80-211 Gdansk, Poland; edyta.dabrowska@gumed.edu.pl (E.D.); ola.michnowska@gmail.com (A.M.); dalia6304@gmail.com (D.T.); lupus@gumed.edu.pl (J.W.); knark@gumed.edu.pl (K.N.); 3 Department of Cardiac Diagnostics, Medical University of Gdansk, 80-211 Gdansk, Poland; marcin.hellmann@gumed.edu.pl; 4 2nd Department of Radiology, Medical University of Gdansk, 80-211 Gdansk, Poland; akfjankowska@gmail.com (A.J.); kujawamariusz1@gmail.com (M.J.K.); dominik.swieton@gumed.edu.pl (D.Ś.); agata.durawa@gumed.edu.pl (A.D.); edyta.szurowska@gumed.edu.pl (E.S.); 5 Oxford University Hospital, Oxford OX3 9DU, UK; agnieszka.brandt-varma@ouh.nhs.uk; 6 Department of Biostatistics and Translational Medicine, Medical University of Lodz, 90-419 Lodz, Poland; jacek.burzynski@student.umed.lodz.pl (J.B.); jedrzej.chrzanowski@umed.lodz.pl (J.C.); arkadiusz.michalak@umed.lodz.pl (A.M.); 7 Department of Pediatrics, Diabetology, Endocrinology and Nephrology, Medical University of Lodz, 90-419 Lodz, Poland; 8 Department of Hypertension and Diabetology, Medical University of Gdansk, 80-211 Gdansk, Poland

**Keywords:** type 1 diabetes, microcirculation, diabetic angiopathies, carotid intima-media thickness

## Abstract

Endothelial dysfunction appears early in type 1 diabetes (T1D). The detection of the first vascular disturbances in T1D patients is crucial, and the introduction of novel techniques, such as flow-mediated skin fluorescence (FMSF) and adaptive optics retinal camera (Rtx) imaging, gives hope for better detection and prevention of angiopathies in the future. In this study, we aimed to investigate microcirculation disturbances in pediatric patients with T1D with the use of FMSF and Rtx imaging. This research focused especially on the relationship between microvascular parameters obtained in FMSF and Rtx measurements, and the glycemic control evaluated in continuous glucose monitoring (CGM) reports. We observed significantly increased wall thickness (WT) and wall-to-lumen ratio (WLR) values in T1D patients in comparison to the control group. Although we did not observe significant differences between the T1D and control groups in the FMSF results, a trend toward significance between the time in range (TIR) and hyperemic response (HR_max_) and an interesting correlation between the carotid intima-media thickness (cIMT_max_) and HR_max_. were observed. In conclusion, FMSF and Rtx measurments are innovative techniques enabling the detection of early microvascular disturbances.

## 1. Introduction

Type 1 diabetes (T1D) is a progressive autoimmune disorder leading to the development of micro- and macrovascular complications. The current T1D therapy with the use of modern technologies has led to a reduction in the incidence rate of angiopathies; however, they consistently remain the major factor responsible for death in patients with T1D diagnosed in childhood. The International Society for Pediatric and Adolescent Diabetes (ISPAD) underlines the importance of screening for T1D complications in the pediatric population [1]. It is essential to treat and prevent the further progression of angiopathies; however, it should be emphasized that only the earliest vascular abnormalities may be reversible [2]

Endothelial dysfunction appears early in type 1 diabetes (T1D) and precedes the diagnosis of T1D complications [3]. At this stage, it remains clinically silent and cannot be revealed in the routine screening tests. Therefore, detecting the first vascular disturbances in T1D patients is crucial. Researchers have put tremendous effort into developing an effective method that could enable the diagnosis and monitoring of endothelial dysfunction. These novel technologies give hope for better prevention of angiopathies in the future. Recently, flow-mediated skin fluorescence (FMSF) and the adaptive optics retinal camera (Rtx), two cutting-edge techniques discussed in the literature, have been used to detect the very first perturbations in microcirculation [4,5,6].

FMSF is a novel, non-invasive tool that is useful in numerous conditions. Previous studies showed that FMSF detected microcirculation disturbances in diabetes [7,8], coronary artery disease [4,9,10], coronavirus disease 2019 [11,12], sleep apnea [13], or even psoriasis [14]. FMSF assesses the vascular endothelial function by monitoring the fluorescence (Fl) of dihydronicotinamide adenine dinucleotide (NADH), which has a crucial role in cellular respiration. The NADH concentration changes in response to the brachial artery occlusion. Thus, the FMSF technique enables the evaluation of the microcirculation function in the post-occlusion period, as well as monitoring the biochemical processes occurring in the tissues during controlled ischemia.

Rtx imaging is based on en face reflectance imaging. It provides high-resolution images close to the histological scale, free from motion distortion. Rtx imaging enables the visualization of the walls of small blood vessels and single retinal photoreceptors non-invasively [15]. Previous studies have already shown that this novel tool is highly effective in the diagnosis of the first vascular changes in diabetic retinopathy (DR) [5,15,16,17]. However, the technology has not yet been tested in children with T1D.

In this study, we aimed to investigate microvascular disturbances in pediatric patients with T1D using FMSF and Rtx imaging. We focused especially on the relationship between the FMSF and Rtx parameters and the glycemic control evaluated in continuous glucose monitoring (CGM) reports. An additional aim was to evaluate the connection between microvascular and macrovascular complications. The correlation between the carotid intima-media thickness (cIMT), as a marker of subclinical atherosclerosis, and microcirculation abnormalities in Rtx and FMSF results was also analyzed.

## 2. Materials and Methods

### 2.1. Study Group

Sixty patients affected with T1D and thirty-nine healthy volunteers (control group), matched by age and sex, were enrolled in the study. The patients were under the care of the Department of Pediatrics, Diabetology, and Endocrinology at the University Clinical Center in Gdansk, Poland. The participants were Caucasian and of Polish origin. The inclusion criteria for the T1D group were as follows: T1D diagnosed according to the ISPAD criteria [18], aged 8–18 years, receiving continuous subcutaneous insulin infusion via an insulin pump for at least 6 months prior to the study, and the willingness to wear a CGM device. The exclusion criteria included diabetic ketoacidosis at the moment of recruitment to the study, ongoing infection, untreated celiac disease, hypothyroidism or other endocrine disorders, chronic respiratory and renal diseases, neurological and immunological disorders, strabismus and nystagmus, multiple daily insulin injections via pen, and the refusal to use a CGM device.

The control group consisted of healthy children without any chronic illnesses. The participants were not on regular medication. The children were recruited from schools and general pediatric clinics.

Before enrolling in the study, all participants underwent a full pediatric review to exclude children with acute health conditions and illnesses seven days beforehand. All patients had their weight and height checked, and the BMI percentile was evaluated using OLAF percentile charts for the Polish pediatric population [19]. Subsequently, blood samples were taken from every individual, and FMSF, Rtx, and cIMT examinations were carried out. All children and families were trained on how to use CGM, and the first CGM sensor was placed during the visit.

This research was performed according to the guidelines of the Declaration of Helsinki. Approval was obtained from the Independent Bioethics Committee for Scientific Research at the Medical University in Gdansk, Poland (No. 746-418/2021). We acquired informed written consent from all of the individuals and their parents or legal guardians.

### 2.2. Laboratory Tests

All participants had biochemical tests, including a full blood count, C-reactive protein and lipid profiles, and kidney and liver function tests, as well as immunological screening for concomitant diseases (autoimmune thyroiditis and celiac disease). A general urinalysis and measurements of the urine albumin/creatinine ratio (UACR) were carried out. The laboratory tests were performed at the University Clinical Center in Gdansk in the certified Central Laboratory.

### 2.3. Flow-Mediated Skin Fluorescence (FMSF)

#### 2.3.1. Technical Principles of FMSF Method

The microvascular function was assessed using FMSF. FMSF is a non-invasive optical technique used to evaluate microcirculation and metabolic regulation based on measurements of the NADH fluorescence intensity in the epidermis. The quantification of the FMSF results was performed using AngioExpert, created by Angionica Ltd. (Lodz, Poland), as described earlier [9,14].

The excitation of the ventral, glabrous side of the forearm with ultraviolet (UV) light at 340 nm results in the emission of an NADH fluorescence signal from the skin tissue cells. The level of NADH fluorescence corresponds to the balance of mitochondrial oxidation–reduction processes occurring in the tissue, reflected by the balance between the oxidized form of the coenzyme (NAD^+^) and its reduced form (NADH). Indeed, NADH fluorescence is the strongest component of the fluorescence emitted from the human skin. The intensity of the signal also changes as a function of time in response to the blockage and release of blood in the brachial artery [12].

The FMSF device consists of three main parts: a light source, a system of filters, and a detector. The UV diode emits light at the 340 nm wavelength and a small amount of blue light to show that the diode is working (Marktech Optoelectronics MTE340H21-UV; peak wavelength: 340 nm; spectral line half width: 9 nm). The blue light is cut through a band pass filter (Hoya U340) that allows it to transmit only UV light at 340 nm and block visible light. Then, the light beam, which has excellent transmission ability through the skin (over 90%), passes through a quartz window.

The fluorescence light at 460 nm emitted by NADH Is detected by the receiver diode (OSI Optoelectronics UV-035EQ). There are 2 filters in front of the detector that block the possibility of light reflected from the hand or the measuring head components reaching the UV detector. The first filter is made of the material Thermoset ADC (CR-39^®^, Edmund Optics), and the second is an interference 460 nm filter (full-width at half-maximum (FWHM): 10 nm; minimum transmission (%): >50; Edmund Optics) [12].

#### 2.3.2. Implementation of the FMSF Technique in the Present Study

Each patient was examined in a sitting position, in a room with a controlled temperature (24 ± 1 °C). After a 15 min acclimatization period, during the first 3 min, resting NADH fluorescence values emitted from the epidermis of the forearm were registered. Then, the sphygmomanometer cuff was pumped 50 mmHg above the systolic pressure to make a brachial artery occlusion. During the 3 min occlusion, the increase in NADH fluorescence was monitored, and the ischemic response (IR) was registered. It was expressed as an IR_max_ (Fl_max_ − Fl_base_/Fl_base_) parameter. Subsequently, the occlusion was released, leading to a decrease in fluorescence, and the hyperemic response (HR) parameters, HR_max_ (Fl_base_ − Fl_min_/Fl_base_) and HR_index_, were measured. HR_max_ reflects the efficacy of the oxygen supply during hyperemia, whereas HR_index_ measures the metabolic recovery after hyperemia [7,10,12]. The other analyzed parameters included the hypoxia sensitivity (HS) and reactive hyperemia response (RHR). HS results from myogenic oscillations at the reperfusion line. RHR (Fl_max_ − Fl_min_/Fl_base_) is the sum of IR_max_ and HR_max_ and depends on the endothelial function in response to nitric dioxide [12]. The fluorescence of the NADH was registered continuously for about 15 min.

### 2.4. Adaptive Optics Retinal Camera (Rtx)

The retinal arterioles were visualized with the use of the adaptive optics retinal camera (Rtx1™; Imagine Eyes, Orsay, France). This camera allows for very precise assessments of microvasculature at a very high resolution due to the use of adaptive optics technology derived from astronomical telescopes, which reduces the effect of atmospheric turbulence during celestial body observations [20]. The device consists of three main elements: a high-resolution fundus camera, a Shack–Hartmann wavefront sensor, and a deformable mirror for the real-time correction of the aberrations of the ocular wavefront [5,21,22]. A beam of light enters the eye, and a small amount is reflected out of the eye and into the optical system [21]. Distortions in light waves caused by irregular optical defects in the eye are eliminated by a deformable mirror, enabling the achievement of high-resolution images [5,15,21,22,23]. Rtx1™ uses en face reflectance imaging with flashed, non-coherent near-infrared illumination [5]. The measurements were taken after 15 min of rest in a sitting position in a dark room. Pupil dilation was not needed. The region of interest included a segment of the superotemporal artery of the right eye, free of the presence of focal arterial nicking and arteriovenous crossings [23]. Three measurements were taken, and the arithmetic mean of the measurements was calculated [15]. The examination delivered several metrics achieved due to AO detection software: lumen diameter (LD), thickness of two walls (WT1 and WT2), outer diameter (OD) resulting from two arteriolar walls (WT1 and WT2) plus the LD, WCSA, and WLR. The WCSA and WLR were calculated automatically by the Rtx software (AOimage 3.4). The WCSA is expressed as π/4 × (OD^2^ − LD^2^), whereas the WLR is equal to (WT1 + WT2)/LD [15,24]. Regarding the WT, we analyzed the highest (WT max), lowest (WT min), and mean values of both WT1 and WT2 measurements [15,24].

### 2.5. Carotid Intima-Media Thickness (cIMT)

The Philips Epiq 5 instrument (Philips Ultrasound, Bothell, WA, USA) was used by an experienced radiologist to measure the cIMT. The patient was asked to rest for 5 min and turn their head to the opposite direction of the examined artery, at around 45 degrees. Linear, 12–18 MHz high-frequency probes were applied. The transducer was placed on the common carotid artery at a 90 degree angle. The cIMT complex was measured approximately 1.5–2 cm below the carotid bifurcation, in the end-diastolic phase. The measurements were repeated three times for each artery, and the arithmetic mean was calculated.

### 2.6. Continuous Glucose Monitoring (CGM)

**Figure 1:**
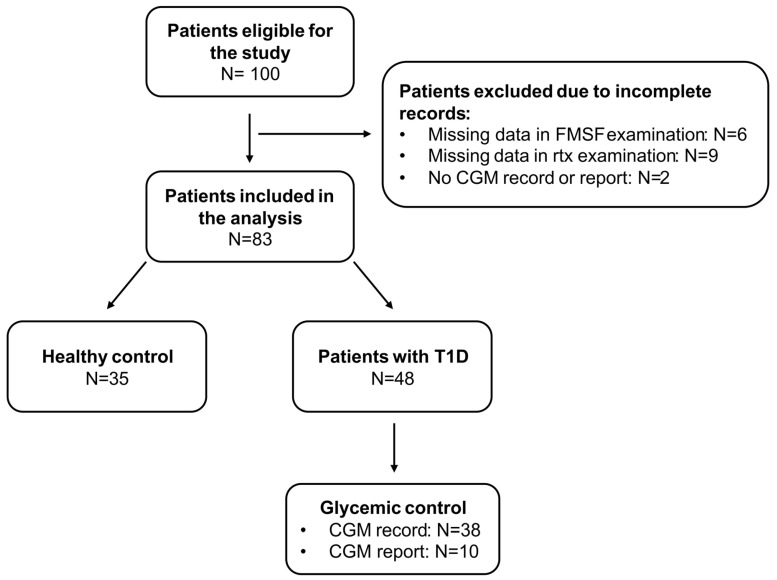
Summary of study group recruitment process. T1D—type 1 diabetes; CGM—continuous glucose monitoring; FMSF—flow-mediated skin fluorescence.

Every patient received a transmitter and three Dexcom G6 sensors for 30 days. We created a Dexcom Clarity account for every patient and asked them to share it with a doctor. The first sensor was placed during the visit. The patients were instructed on how to exchange a sensor. For the majority of the patients with T1D (38 (79.17%)), CGM data were downloaded in .csv format. A further analysis was performed using GlyCulator 3.0. We calculated and analyzed the following indices: mean glucose concentration, coefficient of variation (CV), time spent below range (TBR; <54 mg/dL, <70 mg/dL), time spent in range (TIR; 70–180 mg/dL), and time spent above range (TAR; >180 mg/dL, >250 mg/dL). For 10 (20.83%) patients, we only had access to CGM reports generated using the manufacturer’s platform (Figure 1). We did not detect significant differences in glycemic indices obtained from the manufacturer’s platform and GlyCulator. Only data with record completeness >70% were included in the further analysis. We decided to use a cut-off value of 70% for the TIR, as it constitutes a therapeutic goal in the T1D pediatric population [16].

### 2.7. Statistical Analysis

Continuous variables that displayed non-normal distribution were assessed using the Shapiro–Wilk W test. We reported continuous variables using the median, with 1st and 3rd quartiles. Comparisons between two independent groups were performed using the Mann–Whitney U test. Dichotomous variables were compared using the Chi2 test with appropriate corrections if necessary (Yates test for counts < 15 in at least one group or Fisher’s exact test for counts < 5). We used generalized linear models to adjust the comparisons for diabetes duration. Correlations were assessed with the non-parametric Spearman’s correlation test. The significance threshold alpha was assumed at 0.05. We did not apply a correction for multiple testing. The analyses were performed using Statistica 13.3 (TIBCO, Palo Alto, CA, USA).

## 3. Results

### 3.1. Clinical Characteristics of Patients and CGM Metrics

In total, we recruited 83 subjects, including 35 (42.17%) healthy individuals and 48 (57.83%) diagnosed with T1D. The T1D group consisted of 25 females and 23 males, whereas the control group comprised 18 females and 17 males. The median duration of diabetes was 5.04 (5.18–9.61) years, and their HbA1c content was 7.2% (6.2–7.6%). All patients with T1D wore a CGM device for at least 30 days. Every T1D patient was treated with a CSII and used a Dexcom G6. The median TIR was 63.91 (56.26–74.22%) and was below the therapeutic goal (<70%). The median glycemic variability (CV) rate was slightly above the target of 36% (37.57% (35.77–41.22%)). In total, 14 (29.17%) patients had TIR values above the target value of 70%, and 33 (68.75%) had CV values higher than 36%.

We did not observe statistically significant differences in sex, height, age, or body mass index (BMI) between the study groups. We also did not observe significant differences in thyroid hormone levels, lipid homeostasis, or kidney function. Unsurprisingly, the patients with T1D had significantly higher levels of HbA1c. The study groups did not differ in the presence of comorbidities or additional medication use. Anthropometric data, along with the biochemical results and clinical characteristics, are provided in Table 1.

### 3.2. Comparisons of cIMT, FMSF, and Rtx Examination

**Figure 2:**
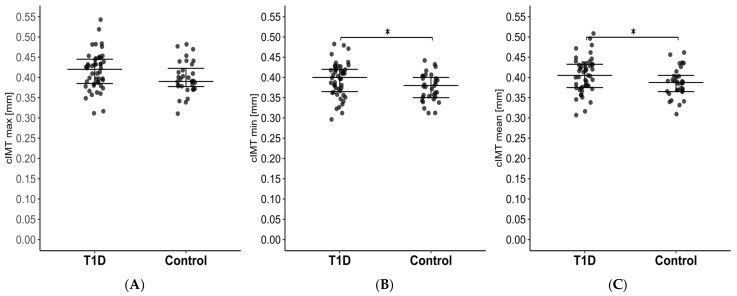
Comparison of carotid intima-media thickness for the (**A**) cIMT_max_, (**B**) cIMT_min_, and (**C**) cIMT_mean_ between patients with T1D and healthy subjects. The middle line displays the median, whereas the whisker spans the lower and upper quantiles. The statistically significant differences are marked with “*”. T1D—type 1 diabetes; cIMT_max_—highest value of carotid intima-media thickness from both artery measurements; cIMT_min_—lowest value of carotid intima-media thickness from both artery measurements; cIMT_mean_—mean value of carotid intima-media thickness from both artery measurements.

We analyzed the maximum (cIMT_max_) and minimum (cIMT_min_) cIMT results, as well as the mean value obtained from both arteries (cIMT_mean_), for every patient. The patients with T1D had significantly higher values for cIMT_mean_ and cIMT_min_ but not cIMT_max_ (Figure 2A–C). We did not notice any significant differences in FMSF parameters (Table 2).

**Figure 3:**
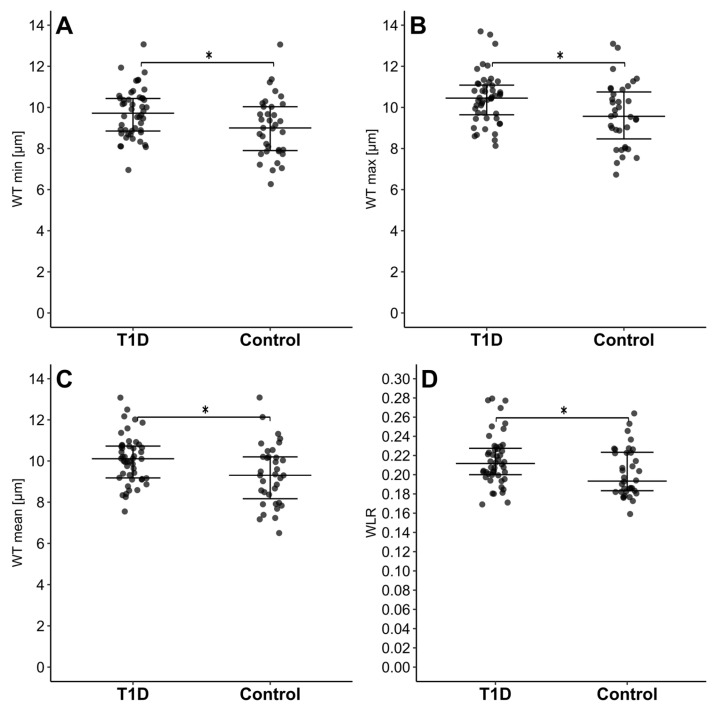
Comparison of adaptive optics retinal camera examination of the (**A**) lowest wall thickness, (**B**) highest wall thickness, (**C**) mean wall thickness, and (**D**) wall-to-lumen ratio results between patients with type 1 diabetes (T1D) and healthy subjects. The middle line displays the median, whereas the whisker spans the lower and upper quantiles. The statistically significant differences between the groups are marked with “*”. T1D—type 1 diabetes; WT_min_—lowest measurement of both wall thicknesses; WT_max_—highest measurement of both wall thicknesses; WT_mean_—mean value from both wall thickness measurements; WLR—wall-to-lumen ratio.

We noticed significantly higher results for the WT_max_, WT_min_, WT_mean_, and wall-to-lumen ratio (WLR) in the patients with T1D (Figure 3A–D). The study groups did not differ regarding their wall cross-section area (WCSA) and arteriolar lumen results. A detailed summary of the cIMT, FMFS, and Rtx results is provided in Table 2.

Given that the microvascular function may be influenced by sex, we decided to perform an adjustment for the patients’ sex. We did not notice a significant difference regarding the primary findings. A summary of the results is provided in Supplementary Tables S1 and S2. Unfortunately, we were not able to evaluate the impact of sexual and pubertal maturity, although these factors may be essential when planning further studies on vascular function.

### 3.3. Relationship Between Glycemic Control and cIMT, FMSF, and Rtx Examinations

**Figure 4:**
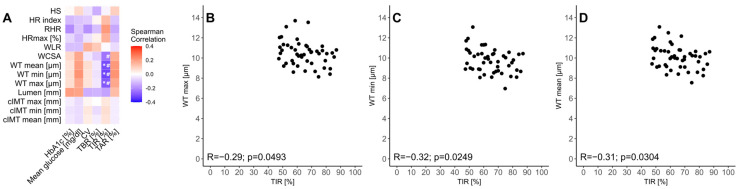
Correlation heatmap (**A**) of glycemic control parameters and carotid intima-media thickness (cIMT), flow-mediated skin fluorescence (FMSF), and adaptive optics retinal camera (Rtx) examinations, as well as scatter plots (**B**–**D**) presenting the relationship between the time in range (70–180 mg/dL) and adaptive optics retinal camera examination results for the (**B**) highest wall thickness, (**C**) lowest wall thickness, and (**D**) mean wall thickness in patients with type 1 diabetes (T1D). Statistically significant correlations are denoted by ‘*’, whereas statistically significant correlations adjusted with T1D duration are marked with ‘^#^’. cIMT_max_—the highest value of carotid intima-media thickness from both artery measurements; cIMT_min_—the lowest value of carotid intima-media thickness from both artery measurements; cIMT_mean_—mean value of carotid intima-media thickness from both artery measurements; HR—hyperemic response; RHR—reactive hyperemia response; HS—hypoxia sensitivity; WT_min_—lowest measurement of both wall thicknesses; WT_max_—highest measurement of both wall thicknesses; WT_mean_—mean value from both wall thickness measurements; WLR—wall-to-lumen ratio; WCSA—wall cross-section area; TIR (70–180)—time in range (70–180 mg/dL); TBR (<70)—time below range (<70 mg/dL); TAR (>180)—time above range (>180 mg/dL); CV—coefficient of variability; HbA1c—hemoglobin A1C.

Finally, we investigated the association of the cIMT, FMSF, and Rtx results in the context of glycemic control from T1D patients. For the Spearman correlations, we observed a significant negative association between the WT_mean_, WT_min_, WT_max_, and TIR (Figure 4A–D). All of these correlations held after adjustment for diabetes duration, uncovering additional significant negative associations between the WCSA and TIR (Figure 4A). Moreover, the patients with a TIR value above 70% had significantly lower levels of WT_max_ (10.27 µm (8.93–10.67 µm) vs. 10.58 µm (9.73–11.27 µm); *p* = 0.0459).

## 4. Discussion

In the present study, we investigated microcirculation disturbances in the T1D pediatric population and their relationship with the glycemic control of the disease. A vast number of studies have already examined microvascular complications in T1D; however, to our knowledge, this was the first time that the FMSF and Rtx techniques were used in the T1D pediatric population.

Our study proved that early microvascular abnormalities develop in childhood, at the early stage of T1D. In addition, we showed that microvascular disturbances correlate with the metabolic control of the disease and confirmed that reaching glycemic targets is essential in T1D therapy after diagnosis. Our results support the previous evidence highlighting the strong correlation between glycemic targets and T1D complications [25].

A minority of the patients met the therapeutic goal of TIR above 70%. Nevertheless, the metabolic control in the present research was better than in the large-cohort studies by Cherubini et al. and Dovc et al. [26,27]. Recent guidelines suggest that the TIR should be ≥80% to achieve optimal metabolic control [25,28]. It is proven that every 10% increase in TIR reduces the risk of DR by 64% and albuminuria by 40% [29].

FMSF seems to have an outstanding diagnostic potential in T1D. It should be emphasized that the FMSF technique reflects all aspects of microvascular blood flow and metabolic regulation. The NOI parameter represents the contribution of endothelial and neurogenic oscillations, whereas the HS parameter reflects the intensity of myogenic oscillations on the reperfusion line [14].

There are no studies regarding the use of FMSF in the pediatric population. The current research revealed some interesting results; however, interpreting the results proved challenging. First of all, we did not observe significant differences between the T1D and control groups. In addition, as many as 21 T1D patients (43.75%) and 17 healthy controls (48.57%) did not reach the baseline fluorescence level after the ischemia–reperfusion cycle. Similarly, Katarzynska et al. observed this phenomenon in a population of young adults with T1D (30–39 years). In this age group, the FMSF parameters were similar between the T1D patients and healthy controls. The authors suggested the existence of a compensatory effect in younger T1D patients that may be linked to erythropoietin stimulation in response to hyperglycemia. On the other hand, this effect was not found in older patients [7]. It remains unclear why some healthy subjects did not reach the baseline fluorescence level. These findings warrant further investigation on a larger group of patients.

The available literature does not contain any data regarding the relationship between FMSF parameters and CGM metrics. Although we did not observe any significant correlations between FMSF and metabolic control in T1D patients, we reported a trend toward significance between the TIR and hyperemic response (HR_max_) after adjusting the *p*-value to the diabetes duration. In addition, we observed higher HR_max_ values in the T1D group with the TIR ≥ 70% in comparison to the group with worse metabolic control; however, this difference did not reach statistical significance. The lower HR_max_ may reflect microvascular endothelial dysfunction. Surprisingly, we did not find any significant correlations between the FMSF parameters and HbA1c. Similarly, Katarzynska et al. did not find a correlation between HR_max_, HR_index,_ and HbA1c; however, it should be underlined that their study group was limited [7]. It should also be noted that HbA1c has several limitations. It reflects the average glucose levels; thus, a person with high glucose variability and improper metabolic control (TIR < 70%) may have the same HbA1c as a patient with stable glucose levels and TIR ≥ 70%. Therefore, CGM metrics are a better metabolic control indicator and should be used widely, not only in clinical practice but also in research.

Furthermore, we found significant differences in numerous Rtx parameters (WT_max_, WT_min,_ WT_mean_, and WLR) between the T1D patients and the healthy individuals. In addition, there was a trend toward significance in the WCSA, whereas the lumen diameter was similar in both groups. This is consistent with previous studies conducted in adult patients with DR. The DR subjects had thicker retinal artery walls, as well as higher WLR and WCSA values, although a difference in lumen diameter was not observed [6,15,30]. On the other hand, Ueno et al. showed lumen narrowing along with the thickening of retinal walls using an Rtx in proliferative DR [31]. It should be emphasized that other retinal imaging techniques corroborate these findings. Stefanski et al. showed higher WLR values in T1D patients with the use of a Heidelberg retina flowmeter [32], and Sampani et al. found an increase in the mean retinal wall thickness and WLR in the T1D group, using adaptive optics scanning laser ophthalmoscopy (AOSLO) [33]. In addition, optical coherence tomography angiography (OCTA) is widely used to detect microvascular retinal changes in T1D [34]. Previous research has proved that both Rtx and optical coherence tomography angiography measurements may be useful in detecting retinal abnormalities at the early stage of diabetes. These techniques are complementary and should be implemented in routine check-ups [6]. However, it is worthwhile noting that only Rtx imaging enables the visualization of retinal vasculature at an almost histological level [35]. The changes observed via vascular wall examinations using Rtx1 incorporate all the layers that build the wall, as Rtx1 does not allow for the differentiation of a particular layer. However, we suspect that wall thickening mainly depends on smooth muscle cell proliferation. According to previous studies on diabetes mellitus that assessed the microvascular structure invasively in subcutaneous gluteal and abdominal tissue, there is a volumetric increase in vascular smooth muscle cells, leading to hypertrophic remodeling [36,37,38]. Optical coherence tomography and scanning laser ophthalmoscopy cannot provide such high-resolution images, mainly due to optical aberrations [35].

The literature regarding the relationship between metabolic control and retinal changes in diabetes is scarce. We reported statistically significant negative correlations between the Rtx parameters (WT_max,_ WT_min_, WT_mean,_ and WCSA) and the TIR. We did not find any studies focusing on T1D metabolic control assessed with CGM metrics or retinal abnormalities detected using an Rtx camera. Researchers have focused on HbA1c to assess metabolic control [24,30]; however, numerous studies in adults showed that CGM metrics are a predictor of the incidence of T1D DR [39,40], and it is well proven that TIR is a better indicator of T1D microvascular complications than HbA1c [41]. Moreover, we found significant correlations between wall thickness values (WT_min_, WT_max_, and WT_mean_) and HbA1c using Rtx imaging, in contrast to the findings of previous studies in the adult population with diabetes [24,30].

The association between micro- and macrovascular complications in diabetes has received much attention in recent years. A study on a vast T1D population from the Diabetes Control and Complication Trial and Epidemiology of Diabetes Interventions and Complications (DCCT/EDIC) revealed the strong relationship between microangiopathy and cardiovascular disease, as well as major adverse cardiovascular events [42]. Although overt signs of macroangiopathy in the pediatric population are rare, it is well known that atherosclerosis develops in childhood. The guidelines recommend the screening of a lipid profile every 3 years and blood pressure control at least annually [1]. cIMT thickening corresponds to subclinical atherosclerosis and is frequently elevated in children with T1D [1,43,44,45,46]. In contrast to our previous study [47], we reported higher cIMT values in T1D individuals in comparison to the control group. On the other hand, we did not observe significant differences in cholesterol or triglyceride concentrations. Interestingly, we reported a negative correlation between the HR_max_ and cIMT, which confirms the relationship between impaired microcirculation and subclinical atherosclerosis, the marker of the first macrovascular changes.

We are aware of the fact that our study has several limitations. Technical issues were the major obstacle we faced during this research. In some cases, it was difficult to provide proper occlusion due to the fact that rigid blood pressure cuffs are not produced in pediatric sizes. In addition, cooperation is crucial in both Rtx and FMSF examinations; thus, we needed time to explain the course of the tests patiently. We were obliged to repeat the information about FMSF for several patients. Occlusion may be uncomfortable for some individuals and may result in dizziness and lightheadedness. Therefore, we assume that FMSF and Rtx measurements may not be effective screening tools in the youngest group of patients of up to 8 years old, especially in individuals with a low arm circumference. In older patients, these techniques seem to be highly reliable; however, in some cases, examinations should be carried out again, preferably on the next day. In addition, the number of participants was limited. Further studies are needed; we plan to continue the study on a larger population and assess the FMSF and Rtx results after two years of follow-up.

The most important strength of the present study is the novelty of the FMSF and Rtx examinations and their possible implementation as screening tools in the future. The preliminary results are very encouraging. Microcirculation disturbances can be observed at the very early stage of the disease using FMSF and Rtx imaging. As the first vascular abnormalities may still be reversible, early detection could not only hinder their progression but also resolve endothelial dysfunction. Moreover, FMSF and Rtx imaging may be helpful in monitoring microvascular changes.

## 5. Conclusions

In conclusion, FMSF and Rtx measurements are innovative techniques enabling the detection of early microvascular disturbances. The severity of the vascular impairment seems to correlate with the metabolic control assessed with the CGM metrics. Our data suggest that Rtx and FMSF measurements could be implemented in clinical practice as screening tools for microangiopathies. They may provide valuable data regarding microcirculation changes in the pediatric population if a child can cooperate during the examination. Given that our study was the first to examine the use of FMSF and Rtx measurements in children with T1D, further investigations are crucial to broadening our knowledge in this area.

## Figures and Tables

**Table 1:** Summary (for continuous variables: median, lower, and upper quartile; for nominal variables: N, %) of anthropometric data and laboratory results.

Variable		T1D N = 48	Control N = 35	*p*-Value
Age [years] (N = 48/N = 35)		13 (11.79–14.96)	13 (11.35–14.97)	0.8141
Body mass [kg] (N = 48/N = 35)		50.65 (38.05–58.50)	48.00 (39.00–56.00)	0.6714
Body mass [z-score] (N = 48/N = 35)		0.15 (−0.42–0.77)	0.16 (−0.43–0.75)	0.9926
Body mass [percentile] (N = 48/N = 35)		55.87 (33.80–77.96)	56.19 (33.51–77.20)	-
Height [cm] (N = 48/N = 35)		162 (152.65–170.55)	160 (150.00–173.00)	0.7433
Height [z-score] (N = 48/N = 35)		0.55 (−0.43–1.07)	0.72 (−0.56–1.51)	0.5928
Height [percentile] (N = 48/N = 35)		70.78 (33.52–85.72)	76.41 (28.83–93.49)	-
BMI [kg/m^2^] (N = 48/N = 35)		18.78 (16.68–20.43)	18.75 (17.48–20.08)	0.8356
BMI [z-score] (N = 48/N = 35)		−0.10 (−0.79–0.50)	−0.17 (−0.82–0.53)	0.9596
BMI [percentile] (N = 48/N = 35)		45.92 (21.35–69.21)	43.43 (20.73–70.14)	-
UACR (N = 48/N = 35)		6.88 (4.17–14.63)	5.60 (0.00–8.47)	0.1457
HbA1c [%] (N = 48/N = 35)		7.20 (6.30–7.60)	5.30 (5.20–5.50)	<0.0001
TSH [uU/mL] (N = 47/N = 35)		1.65 (1.22–2.14)	1.58 (1.27–2.08)	>0.9999
FT4 [pmol/L] (N = 47/N = 34)		11.60 (11.07–12.65)	11.34 (10.69–12.74)	0.4298
TC [mg/dL] (N = 46/N = 35)		157.00 (139.00–170.00)	157.00 (144.00–178.00)	0.6817
LDL-C [mg/dL] (N = 46/N = 35)		88.50 (73.00–102.00)	90.00 (78.00–99.00)	0.9392
HDL-C [mg/dL] (N = 46/N = 35)		57.50 (53.00–63.00)	55.00 (49.00–63.00)	0.2521
TG [mg/dL] (N = 46/N = 35)		52.00 (44.00–60.00)	58.00 (40.00–73.00)	0.2855
Sex	Female	25 (52.08%)	18 (51.43%)	0.9529
	Male	23 (47.92%)	17 (48.57%)	
Celiac disease	Yes	3 (6.25%)	1 (2.86%)	0.6348
	No	45 (93.75%)	34 (97.14%)	
Autoimmune thyroiditis	Yes	5 (10.42%)	0 (0.00%)	0.0765
	No	43 (89.58%)	35 (100%)	
Albuminuria (UACR > 30 mg/g)	Yes	12 (25.00%)	4 (11.43%)	0.1624
	No	36 (75.00%)	31 (88.57%)	

BMI—body mass index; UACR—urine albumin/creatinine ratio; HbA1c—hemoglobin A1C; TSH—thyroid stimulating hormone; FT4—thyroxine; TC—total cholesterol; TG—triglycerides; LDL-C—low-density lipoprotein cholesterol; HDL-C—high-density lipoprotein cholesterol.

**Table 2:** Summary (median, lower, and upper quantiles) of carotid intima-media thickness (cIMT), flow-mediated skin fluorescence (FMSF), and adaptive optics retinal camera (Rtx) examinations.

Variable	T1D N = 48	Control N = 35	*p*-Value
cIMT_min_ [mm] (N = 47/N = 32)	0.40 (0.36–0.42)	0.37 (0.34–0.40)	0.0278
cIMT_max_ [mm] (N = 47/N = 32)	0.42 (0.38–0.45)	0.39 (0.37–0.42)	0.0856
cIMT_mean_ [mm] (N = 47/N = 32)	0.41 (0.38–0.44)	0.39 (0.37–0.41)	0.0472
HR_max_ [%](N = 48/N = 35)	17.80 (15.20–21.10)	19.75 (17.48–21.48)	0.1388
RHR (N = 48/N = 35)	28.25 (17.55–37.65)	27.31 (20.15–37.79)	0.7574
HR_index_ [%](N = 48/N = 35)	11.15 (8.30–12.80)	11.09 (8.76–12.01)	0.9963
HS (N = 48/N = 35)	102.45 (54.10–164.40)	80.44 (42.13–156.81)	0.5520
Lumen [μm] (N = 48/N = 35)	96.00 (90.25–102.08)	95.67 (83.60–101.47)	0.5428
WT_min_ [μm] (N = 48/N = 35)	9.72 (8.83–10.43)	9.00 (7.87–10.03)	0.0187
WT_max_ [μm] (N = 48/N = 35)	10.45 (9.58–11.10)	9.57 (8.07–10.87)	0.0149
WT_mean_ [μm] (N = 48/N = 35)	10.11 (9.17–10.73)	9.30 (7.97–10.20)	0.0189
WCSA (N = 48/N = 35)	3346.76 (2958.05–3756.59)	3116.53 (2601.94–3490.76)	0.0774
WLR (N = 48/N = 35)	0.21 (0.20–0.23)	0.19 (0.18–0.22)	0.0326

cIMT_max_—highest value of carotid intima-media thickness from both artery measurements; cIMT_min_—lowest value of carotid intima-media thickness from both artery measurements; cIMT_mean_—mean value of carotid intima-media thickness from both artery measurements; HR—hyperemic response; RHR—reactive hyperemia response; HS—hypoxia sensitivity; WT_min_—lowest measurement of both wall thicknesses; WT_max_—highest measurement of both wall thicknesses; WT_mean_—mean value from both wall thicknesses measurements; WLR—wall-to-lumen ratio; WCSA—wall cross-section area.

## Data Availability

The data presented in this study are available on request from the corresponding author. The data are not publicly available due to privacy reasons.

## Supplementary Materials

The following supporting information can be downloaded at https://www.mdpi.com/article/10.3390/bios15070439/s1: Figure S1. An example of NADH fluorescence traces of a patient with type 1 diabetes in response to the blockage and release of the blood flow in the brachial artery.; Figure S2. Images of the retinal artery in a patient with type 1 diabetes captured by the retinal adaptive optics camera (rtx; Imagine Eyes, Orsay, France). Table S1. Summary (median, lower, and upper quantiles) of carotid intima-media thickness (cIMT), flow-mediated skin fluorescence (FMSF), and adaptive optics retinal camera (Rtx) examinations regarding the patient’s sex. Table S2. Summary (median, lower, and upper quantiles) of carotid intima-media thickness (cIMT), flow-mediated skin fluorescence (FMSF), and adaptive optics retinal camera (Rtx) examinations with adjustment for patient’s sex.

## Abbreviations

The following abbreviations are used in this manuscript: BMI—body mass index; CGM—continuous glucose monitoring; cIMT_max_—highest value of carotid intima-media thickness from both artery measurements; cIMT_min_—lowest value of carotid intima-media thickness from both artery measurements; cIMT_mean_—mean value of carotid intima-media thickness from both artery measurements; CV—coefficient of variability; FMSF—flow-mediated skin fluorescence; FT4—thyroxine; HbA1c—hemoglobin A1C; HDL-C—high-density lipoprotein cholesterol; HR—hyperemic response; HS—hypoxia sensitivity; IR—ischemic response; LDL-C—low-density lipoprotein cholesterol; NADH—dihydronicotinamide adenine dinucleotide; Rtx—retinal adaptive optics camera; T1D—type 1 diabetes; TAR (>180)—time above range (>180 mg/dL); TBR (<70)—time below range (<70 mg/dL); TC—total cholesterol; TG—triglycerides; TSH—thyroid-stimulating hormone; TIR (70–180)—time in range (70–180 mg/dL); WCSA—wall cross-section area; WLR—wall-to-lumen ratio; RHR—reactive hyperemia response; WT1—thickness of first retinal arteriole wall; WT2—thickness of second retinal arteriole wall; WT_min_—lowest measurement of both wall thicknesses; WT_max_—highest measurement of both wall thicknesses; WT_mean_—mean value of both wall thickness measurements; UACR—urine albumin/creatinine ratio.
