# Supplementary material for: Assessment of Microvascular Disturbances in Children with Type 1 Diabetes—A Pilot Study
Source: Biosensors (Basel). 2025 Jul 8;15(7):439. doi: 10.3390/bios15070439 (PMC12293879; doi:10.3390/bios15070439)
Supplement: Supplementary file 1 [file biosensors-15-00439-s001.zip › biosensors-3550835-supplementary.pdf]

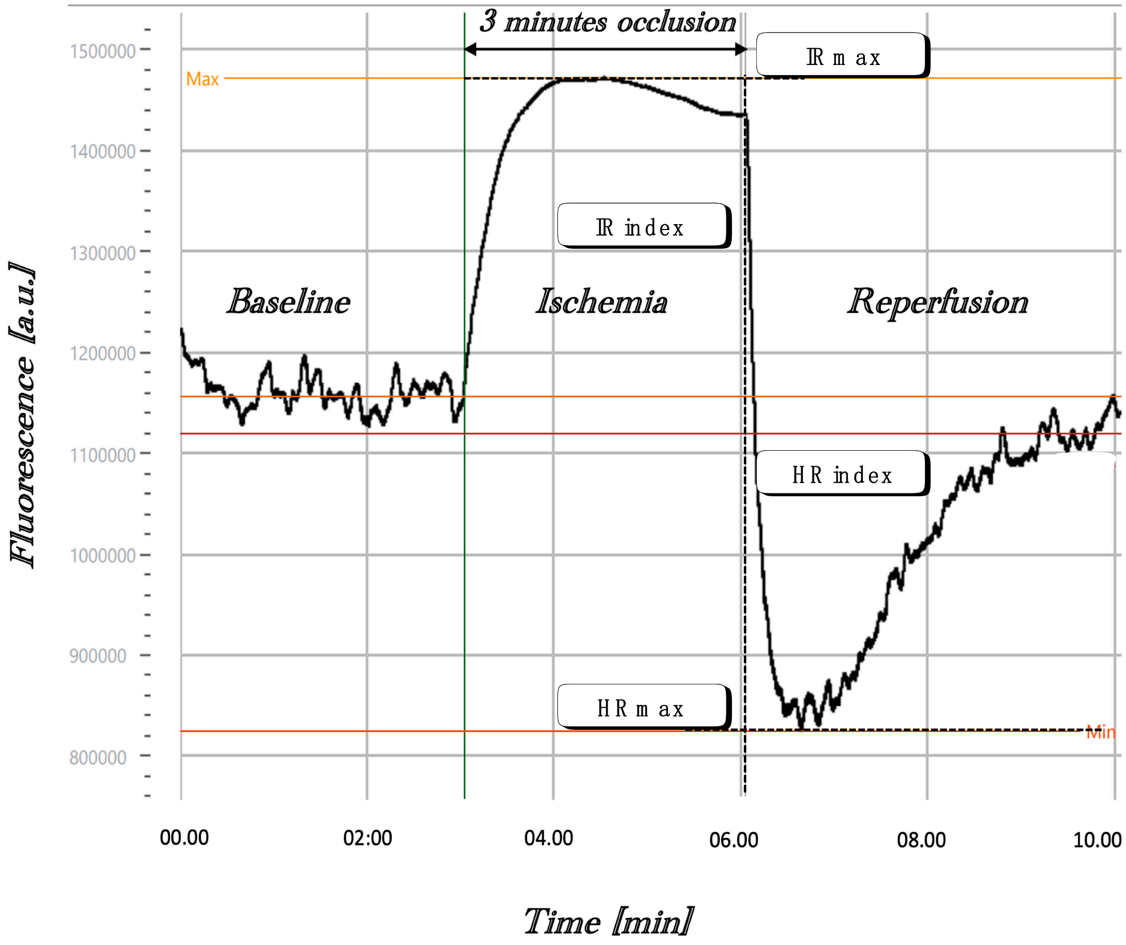

Figure S1. An example of NADH fluorescence traces of a patient with type 1 diabetes in response to the blockage and release of the blood flow in the brachial artery. The rapid increase in NADH concentration is visible during ischemia, followed by the fall of the NADH and a slow recovery to the baseline concentration. The fluorescence was measured with the use of flow-mediated skin fluorescence (FMSF; Angiogenica, Lodz, Poland).

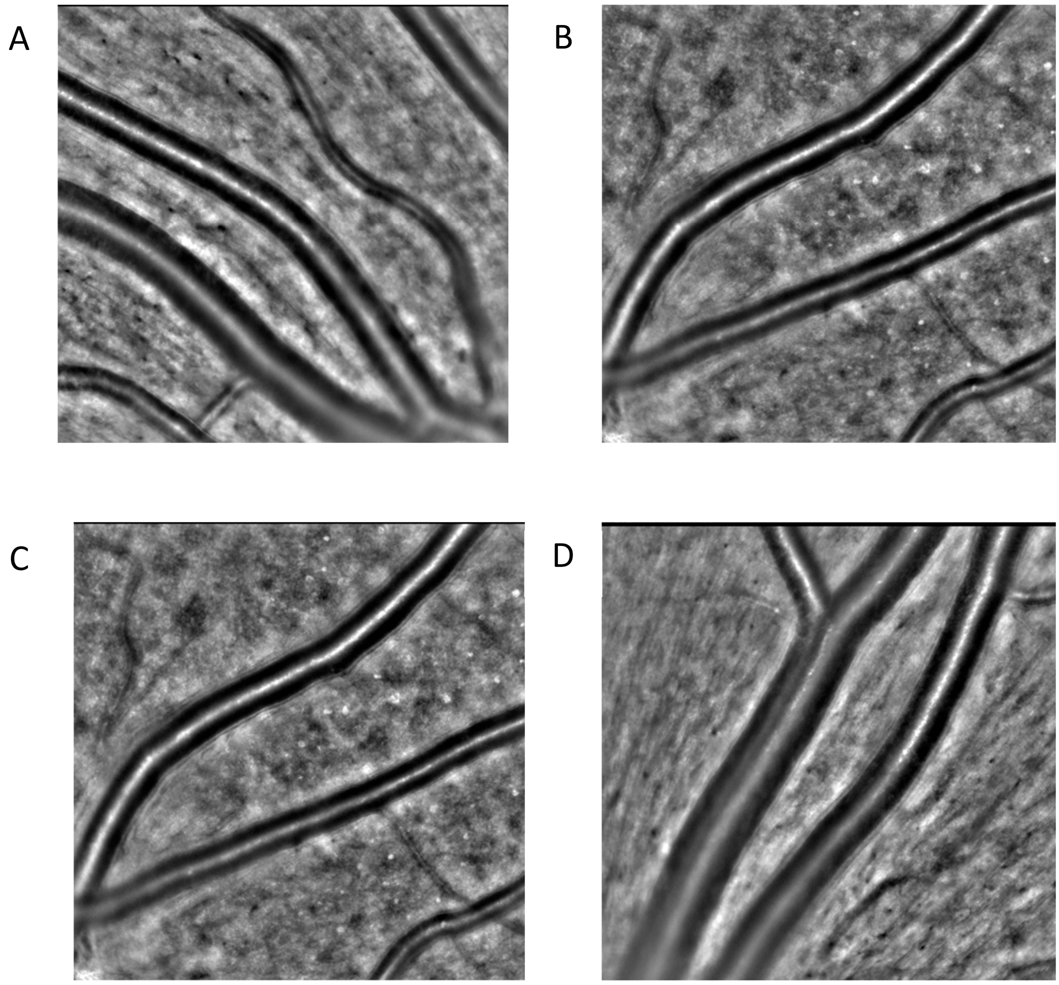

Figure S2. Images of the retinal artery in a patient with type 1 diabetes captured by the retinal adaptive optics camera (rtx; Imagine Eyes, Orsay, France).

Table S1. Summary (median, lower, and upper quantiles) of carotid intima-media thickness (cIMT), flow-mediated skin fluorescence (FMSF), and adaptive optics retinal camera (Rtx) examinations regarding the patient's sex.

Variable	Me (Q1-Q3)		p-value
	Female (N=43)	Male (N=40)	
**Age [lata]**	13 (11.53 - 14.84)	13 (11.77 - 14.65)	0.9855
**Body mass [kg]**	50.70 (36.55 - 55.40)	47.00 (39.75 - 60.42)	0.5175
**Body mass [z-score]**	0.16 (-0.35 - 0.71)	0.15 (-0.6 - 0.81)	0.9165
**Body mass [percentile]**	56.19 (36.28 - 76.03)	55.96 (27.53 - 79.05)	-
**Height [cm]**	162.00 (150.00 - 169.50)	161.00 (151.88 - 173)	0.7428
**Height [z-score]**	0.71 (-0.43 - 1.45)	0.46 (-0.49 - 1.14)	0.2307
**Height [percentile]**	76.15 (33.31 - 92.65)	67.8 (31.23 - 87.32)	
**BMI [kg/m2]**	18.67 (16.47 - 20.24)	18.77 (17.29 - 20.41)	0.4332

**BMI [z-score]**	-0.16 (-0.84 - 0.29)	-0.05 (-0.54 - 0.58)	0.3070
**BMI [percentile]**	43.73 (20.13 - 61.21)	48.07 (29.63 - 71.97)	-
**Tanner scale**	3 (2-4)	3 (2-4)	0.3993
**cIMT [mm]**	0.39 (0.37 - 0.42)	0.41 (0.38 - 0.44)	0.0817
**$cIMT_{min}$ [mm]**	0.38 (0.35 - 0.41)	0.40 (0.36 - 0.42)	0.0864
**$cIMT_{max}$ [mm]**	0.40 (0.38 - 0.43)	0.43 (0.38 - 0.45)	0.0908
**HR_{max}[%]**	18.13 (14.55 - 20.17)	19.35 (17.5 - 22.12)	**0.0464**
**RHR**	25.90 (18.56 - 36.85)	31.4 (19.66 - 41.46)	0.3095
**HR_{index}[%]**	10.60 (8.25 - 12.33)	11.25 (9.36 - 12.86)	0.1863
**HS**	105.60 (48.45 - 185.75)	73.48 (46.67 - 143.12)	0.2048
**WT_{max} [μm]**	9.73 (9.00 - 10.65)	10.48 (9.46 - 11.28)	**0.0301**
**WT_{min} [μm]**	9.17 (8.40 - 10.03)	9.85 (8.72 - 10.62)	**0.0412**
**WT_{mean} [μm]**	9.60 (8.68 - 10.20)	10.14 (9.14 - 10.93)	**0.0294**
**WCSA**	3199.51 (2761.90 - 3548.37)	3328.04 (2847.92 - 3900.07)	0.1858
**WLR**	0.20 (0.18 - 0.22)	0.22 (0.20 - 0.23)	**0.0263**

BMI—Body Mass Index; $cIMT_{max}$—higher value of carotid intima-media thickness from both artery measurements; $cIMT_{min}$—lower value of carotid intima-media thickness from both artery measurements; $cIMT_{mean}$—mean value of carotid intima-media thickness from both artery measurements; HR—hyperemic response; RHR—reactive hyperemia response; HS—hypoxia sensitivity; WT_{min}—lower measurement of both wall thicknesses; WT_{max}—higher measurement of both wall thicknesses; WT_{mean}—mean value from both wall thicknesses measurements; WLR—wall-to-lumen ratio; WCSA—wall cross-section area.

Table S2. Summary (median, lower, and upper quantiles) of carotid intima-media thickness (cIMT), flow-mediated skin fluorescence (FMSF), and adaptive optics retinal camera (Rtx) examinations with adjustment for patient's sex.

Variable	Me (Q1-Q3)		p-value	p-value adjsuted for sex
	T1D (N=48)	Control (N-35)		
**$cIMT_{min}$ [mm]**	0.40 (0.36—0.42)	0.37 (0.34—0.40)	**0.0278**	**0.0313**
**$cIMT_{max}$ [mm]**	0.42 (0.38-0.45)	0.39 (0.37—0.42)	0.0856	0.0910
**$cIMT_{mean}$ [mm]**	0.41 (0.38 - 0.44)	0.39 (0.37 - 0.41)	**0.0472**	**0.0490**
**HR_{max} [%]**	17.80 (15.20 - 21.10)	19.75 (17.48 - 21.48)	0.1388	0.0871
**RHR**	28.25 (17.55 - 37.65)	27.31 (20.15 - 37.79)	0.7574	0.7328

**HR_{index} [%]**	11.15 (8.30 - 12.80)	11.09 (8.76 - 12.01)	0.9963	0.5376
**HS**	102.45 (54.10 - 164.40)	80.44 (42.13 - 156.81)	0.5520	0.8028
**Lumen [μm]**	96.00 (90.25 - 102.08)	95.67 (83.60 - 101.47)	0.5428	0.4577
**WT_{min} [μm]**	9.72 (8.83 - 10.43)	9.00 (7.87 - 10.03)	**0.0187**	**0.0199**
**WT_{max} [μm]**	10.45 (9.58 - 11.10)	9.57 (8.07 - 10.87)	**0.0149**	**0.0113**
**WT_{mean} [μm]**	10.11 (9.17 - 10.73)	9.30 (7.97 - 10.20)	**0.0189**	**0.0129**
**WCSA**	3 346.76 (2 958.05 - 3 756.59)	3 116.53 (2 601.94 - 3 490.76)	0.0774	0.0534
**WLR**	0.21 (0.20 - 0.23)	0.19 (0.18 - 0.22)	**0.0326**	**0.0316**

$cIMT_{max}$—higher value of carotid intima-media thickness from both artery measurements; $cIMT_{min}$—lower value of carotid intima-media thickness from both artery measurements; $cIMT_{mean}$—mean value of carotid intima-media thickness from both artery measurements; HR—hyperemic response; RHR—reactive hyperemia response; HS—hypoxia sensitivity; WT_{min}—lower measurement of both wall thicknesses; WT_{max}—higher measurement of both wall thicknesses; WT_{mean}—mean value from both wall thickness measurements; WLR—wall-to-lumen ratio; WCSA—wall cross-section area.
